# Modulation of Dental Pulp Stem Cell Odontogenesis in a Tunable PEG-Fibrinogen Hydrogel System

**DOI:** 10.1155/2015/525367

**Published:** 2015-06-01

**Authors:** Qiqi Lu, Mirali Pandya, Abdul Jalil Rufaihah, Vinicius Rosa, Huei Jinn Tong, Dror Seliktar, Wei Seong Toh

**Affiliations:** ^1^Faculty of Dentistry, National University of Singapore, 11 Lower Kent Ridge Road, Singapore 119083; ^2^Department of Surgery, Yong Loo Lin School of Medicine, National University Health System, National University of Singapore, 1E Kent Ridge Road, Singapore 119288; ^3^Nanoscience and Nanotechnology Initiative, Faculty of Engineering, National University of Singapore, 2 Engineering Drive 3, Singapore 117581; ^4^Faculty of Biomedical Engineering, Technion-Israel Institute of Technology, 32000 Haifa, Israel; ^5^Tissue Engineering Program, Life Sciences Institute, National University of Singapore, 27 Medical Drive, Singapore 117510

## Abstract

Injectable hydrogels have the great potential for clinical translation of dental pulp regeneration. A recently developed PEG-fibrinogen (PF) hydrogel, which comprises a bioactive fibrinogen backbone conjugated to polyethylene glycol (PEG) side chains, can be cross-linked after injection by photopolymerization. The objective of this study was to investigate the use of this hydrogel, which allows tuning of its mechanical properties, as a scaffold for dental pulp tissue engineering. The cross-linking degree of PF hydrogels could be controlled by varying the amounts of PEG-diacrylate (PEG-DA) cross-linker. PF hydrogels are generally cytocompatible with the encapsulated dental pulp stem cells (DPSCs), yielding >85% cell viability in all hydrogels. It was found that the cell morphology of encapsulated DPSCs, odontogenic gene expression, and mineralization were strongly modulated by the hydrogel cross-linking degree and matrix stiffness. Notably, DPSCs cultured within the highest cross-linked hydrogel remained mostly rounded in aggregates and demonstrated the greatest enhancement in odontogenic gene expression. Consistently, the highest degree of mineralization was observed in the highest cross-linked hydrogel. Collectively, our results indicate that PF hydrogels can be used as a scaffold for DPSCs and offers the possibility of influencing DPSCs in ways that may be beneficial for applications in regenerative endodontics.

## 1. Introduction

In recent years, there has been an increasing focus on conservative strategies in endodontics for the treatment of diseased dental pulps. In the emerging paradigm of regenerative endodontics, stem cell and tissue engineering technologies offer potential for repair and regeneration of dentin-pulp tissues to treat necrotic teeth in patients [1, 2].

Dental pulp stem cells (DPSCs) have been demonstrated as a suitable cell source for dental tissue regeneration, owing to their high accessibility, proliferative ability, and multilineage differentiation potential [3, 4]. Inductive biochemical factors for odontoblastic differentiation of DPSCs have been well studied in recent years [5–7]. However, there is still limited understanding of the ideal scaffold design and the critical stem-cell biomaterial interactions that are needed to support DPSCs to regenerate dental tissues. Of note, the mechanical properties of the artificial extracellular microenvironment and how they may affect the behavior of DPSCs are still poorly understood.

In the context of dental pulp tissue engineering, the use of injectable hydrogels as scaffolds is particularly attractive as they are expected to conform to the variable shape of the pulp chamber and can be formulated with cells and/or growth factors by simple mixing [8–12]. We have previously developed an injectable semisynthetic hydrogel scaffold material comprised of a fibrinogen backbone covalently conjugated to polyethylene glycol (PEG) side chains and cross-linked by photopolymerization. The hydrogel's mechanical properties may also be tuned by the degree of cross-linking through a simple adjustment of the concentration of additional PEG-diacrylate (PEG-DA), photoinitiator, and ultraviolet (UV) light intensity [13]. Furthermore, the unique photo-cross-linking property of the hydrogel would allow dentists to inject the precursor solution into the dental pulp chamber before rapid gelation by photopolymerization.

In this study, we examined the cytocompatibility of PEG-fibrinogen (PF) hydrogels and the effects of varying the hydrogel cross-linking degrees on the differentiation of encapsulated dental pulp stem cells (DPSCs). We hypothesized that hydrogels of varying cross-linking degrees might induce cellular morphological changes and impact on the extent of differentiation. We also hypothesized that PF hydrogels with a higher cross-linking degree and correspondingly higher storage modulus would facilitate odontoblastic differentiation of DPSCs. This study may prove useful in designing injectable hydrogel scaffolds for applications in dental pulp tissue engineering and regenerative endodontics.

## 2. Materials and Methods

### 2.1. Synthesis of PEG-Fibrinogen Precursors

PEG fibrinogen (PF) was synthesized according to published protocols [13]. Briefly, 7 mg/mL bovine fibrinogen (Bovogen Biologicals Pty Ltd., Australia) was dissolved in 10 mM phosphate buffered saline (PBS) with 8 M urea. Tris(2-carboxyethyl)phosphine hydrochloride (TCEP-HCL) was added to the solution at a molar ratio of 1.5 : 1 to the amount of cysteines in fibrinogen. The fibrinogen solution was then adjusted to pH 8 using sodium hydroxide (NaOH). The PEG-DA solution was prepared by dissolving 280 mg/mL PEG-DA (linear, 10 kDa) powder in 10 mM PBS with 8 M urea. After centrifugation, the supernatant containing dissolved PEG-DA was added to the fibrinogen solution at a 3.7 : 1 molar ratio of PEG to fibrinogen cysteines. The mixture was then reacted in a reaction vessel with a thermostatic jacket (Lenz Laborglas, Germany) at 22.5°C for 3 h in the dark. After reaction, the solution was first diluted with equal amount of PBS containing 8 M urea and then precipitated by adding into acetone at a volume ratio of 4 : 1 (acetone to solution). The precipitant was redissolved by adding 1.8 volumes of PBS-8 M urea. The modified fibrinogen was then purified and concentrated to 8–12 mg/mL by using Centramate cassettes (50 kDa MW cutoff, NY, USA). The PEG-fibrinogen (PF) solution was subsequently passed through a high shear fluid processor (Microfluidics M110-P, USA) to achieve uniform particle size and finally filtered with VacuCap 90 filter (Pall Corporation, USA).

### 2.2. Fabrication of PF Hydrogels

PF hydrogels with different cross-linking degrees were made by cross-linking PF gels using variable amount of additional PEG-DA. Four groups containing 0, 0.5, 1.5, and 2.5% w/v additional PEG-DA were prepared. To make these gels, a precursor solution containing four components was prepared: (1) PF hydrogel (final concentration 8 mg/mL); (2) PEG-DA (diluted from 15% stock solution in PBS); (3) 1% (v/v) photoinitiator solution (prepared from stock solution containing 10% w/v Irgacure 2959 (Ciba, Switzerland) in 70% ethanol); (4) PBS (volume adjustable). The precursor solutions were first mixed by vortexing for 3 s and then transferred to plastic cylinder molds (150 *μ*L, Fisher Scientific) or 15-well *μ*-slides (Ibidi) for gel casting. To initiate cross-linking, the precursor solutions were exposed to long-wave UV light (365 nm, 4-5 mW/cm^2^) for 3 min in 15-well *μ*-slide or 5 min in plastic cylinder molds.

### 2.3. Rheological Characterization of PF Hydrogels

Rheological measurements of PF hydrogels were carried out using AR-G2 rheometer (TA instruments, New Castle, DE, USA) according to a published protocol [14]. Briefly, after 1 min equilibrium time, 200 *μ*L PF precursor solution loaded on the instrument was cross-linked by exposure to long-wave UV light (365 nm) emitted from OmniCure Series 2000 UV light source at an intensity of 5 mW/cm^2^. Time-sweep oscillatory tests were done at room temperature at a sinusoidal 2% strain rate and a 6-rad s^−1^ angular frequency, within the linear viscoelastic region as determined previously [15]. Temporal change of storage modulus (*G*′) was recorded for 3 min after initiation of cross-linking.

### 2.4. Swelling Analysis of PF Hydrogels

For measuring the swelling ratio, PF hydrogels were cast into cylinder molds, transferred to PBS, and allowed to equilibrate for 24 h at 4°C. Wet weight of the samples was determined before undergoing freeze-drying overnight. The change in hydrogel weight between the swollen (*W*_*s*_) and dried (*W*_*d*_) states was used to determine the volumetric swelling ratio as follows:

(1) $$\begin{array}{c} \text{Swelling}\;\text{ratio}=\frac{W_{s}}{W_{d}}. \end{array}$$

### 2.5. DPSC Encapsulation and Differentiation

Human DPSCs used in this study were obtained commercially from AllCells, LLC. DPSCs were cultured in Dulbecco's modified eagle's medium (DMEM) (low glucose, Biowest, France) supplemented with 10% fetal bovine serum (FBS, Biowest) and 1% penicillin/streptomycin (PS, Life technologies, Singapore). To initiate cell encapsulation, DPSCs at P4-P5 were detached by trypsinization before being resuspended in PF gel precursor solution at a density of 1 × 10^6^ cells/mL. To initiate cross-linking, the cell-gel mixtures were exposed to long-wave UV light for 3 min in 15-well *μ*-slide or 5 min in plastic cylinder molds. DPSCs encapsulated in the PF hydrogels were differentiated to odontoblast-like cells by culturing in odontogenic/osteogenic differentiation medium [16] comprised of DMEM high glucose, 10% FBS, 1% PS, 10^−7^ M dexamethasone (Sigma, St. Louis, MO, USA), 50 *μ*g/mL ascorbic acid 2-phosphate (Sigma), and 10 mM *β*-glycerophosphate (Sigma). DPSCs encapsulated in PF hydrogels (cast in both cylinder molds and in 15-well *μ*-slides) were differentiated for 3 weeks with medium change every 3 days.

### 2.6. Cell Viability

Encapsulated DPSCs in PF hydrogels were stained by live/dead viability assay kit (Life Technologies) according to the manufacturer's protocol. Briefly, the medium was removed and the cells in hydrogel were rinsed once with PBS followed by incubation with the dye. Live cells stained green with calcein AM and dead cells marked red with ethidium homodimer-1 (EthD-1) were visualized using a confocal microscope. Two to three random sections were analyzed for each sample (*n* = 2-3 samples per gel type). The cell count was performed using ImageJ software (NIH, Bethesda, MD). Percentage cellular viability was calculated as the number of viable cells is divided by the total number of cells measured per gel, multiplied by 100.

### 2.7. Cell Morphology

After 21 days of culture, DPSC-laden hydrogel constructs were fixed in 10% buffered formalin overnight at 4°C. The PF hydrogel constructs were further cryoprotected in 25% sucrose solution, flash-frozen in isobutane (−30°C), embedded in Tissue-Tek O.C.T compound (Sakura Finetek Inc., Torrance, CA, USA), and cryosectioned at 30 *μ*m [17]. The sections were stained with haematoxylin and eosin (H&E) following the standard histology technique, as previously described [18]. Cell morphology was assessed by analysis of the H&E stained cross-sections of the PF hydrogel constructs. Digital micrographs were taken at 10x objective lens magnification and percentage of cellular aggregation was analyzed using ImageJ software (NIH, Bethesda, MD) and expressed as the percentage of total number of cells, as previously described [19]. Two to three random sections were analyzed for each sample (*n* = 2-3 samples per gel type).

### 2.8. Real-Time RT-PCR

For real-time reverse transcriptase polymerase chain reaction (RT-PCR), PF hydrogels were casted in cylinder molds with 1 × 10^6^/mL DPSCs. Total RNA of DPSCs was extracted by NucleoSpin RNA extraction kit (Macherey-Nagel, Germany). Purified RNA was then transcribed to cDNA by iScript cDNA synthesis kit (Biorad, Hercules, CA, USA). For PCR reaction, 2X iScript one-step RT-PCR reagent (Biorad) was mixed with cDNA templates and primers. The RT-PCR reactions were performed in CFX Connect real-time PCR machine (Biorad) at 95°C for 3 min followed by 40 cycles of 10 s denaturation at 95°C and 30 s annealing at 55°C. For all experiments, glyceraldehyde-3-phosphate dehydrogenase (*GAPDH*) was used as internal reference gene and 3 samples (*n* = 3 samples per gel type) were assigned in each group for quantification. The primer sequences of collagen I (*Col I*), dentin sialophosphoprotein (*DSPP*), dentin matrix protein-1 (*DMP-1*), osteocalcin (*OC*), and* GAPDH* are detailed in Table 1.

### 2.9. Calcium Assay and DNA Quantification

Calcium assay and DNA quantification required lysis of PF hydrogels and the encapsulated DPSCs. After differentiation for 3 weeks, samples were transferred to 1.5 mL microtubes containing PBS with 0.2% Tween 20 (Sigma) for lysis. After incubation at 37°C for 2 h, the lysed samples were stored at −20°C for subsequent assays. For measurements of calcium and DNA amounts, calcium assay (Cayman, Ann Arbor, MI, USA) and PicoGreen dsDNA quantification assay (Life Technologies) were performed, respectively, according to the manufacturers' instructions. Readings were taken using the Infinite 2000 plate reader (Tecan, Austria). The amount of calcium present in each sample was normalized to the corresponding DNA amount.

### 2.10. Statistical Analysis

The data were reported as mean ± standard deviation (SD). Analysis of variance (ANOVA) and Fisher's protected least squares difference (PLSD)* post hoc* testing were performed using StatView software (SAS Institute Inc., Cary, NC, USA). Statistical significance was set as *P* < 0.05.

## 3. Results

### 3.1. Hydrogel Characterization

The amount of additional PEG-DA cross-linker (from 0 to 2.5 wt %) was varied to form PEG-fibrinogen (PF) hydrogels of four different cross-linking degrees (PF-0, PF-0.5, PF-1.5, and PF-2.5), respectively, which spanned a range of mechanical and swelling properties.

The formation of PF hydrogels was evaluated using oscillatory rheometry which measures the shear storage modulus (*G*′) and loss modulus (*G*′′) against the shear strain. The gel point, defined as the crossover of *G*′ and *G*′′, was employed to evaluate the gelation rate of the hydrogel. As summarized in Table 2, the peak values of *G*′ that represented the stiffness of the fully cross-linked hydrogel were tunable by the amount of PEG-DA added to the precursor solutions. The addition of 0, 0.5, 1.5, and 2.5 wt % PEG-DA resulted in mean peak *G*′ values of 140 ± 2, 454 ± 13, 1574 ± 21, and 3601 ± 47 Pa, respectively. This change in *G*′ with increasing PEG-DA percentage proved to be statistically significant (*P* < 0.0001, power = 1). Also, the gel point of the hydrogels ranged from 11.4 to 23.4 s with increasing amount of PEG-DA. In addition, the time required for *G*′ to reach the peak and then plateau also increased significantly with the increase in PEG-DA percentage (*P* < 0.0001, power = 1). These results indicate that the addition of PEG-DA cross-linker allows for a higher degree of PF hydrogel cross-linking that results in higher modulus but also requires longer times for completion of the cross-linking process.

**Figure 1:**
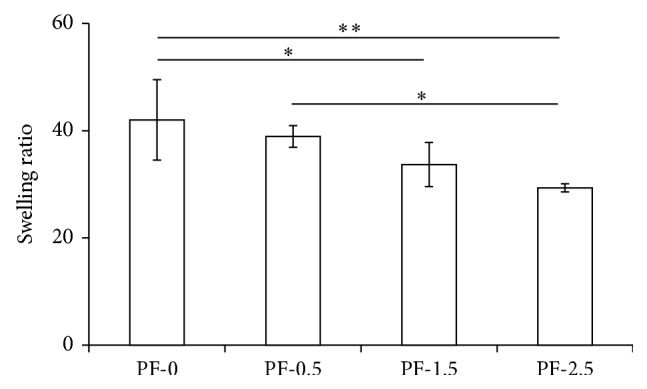
Swelling ratio of PF hydrogels (PF-0, PF-0.5, PF-1.5, and PF-2.5) cross-linked with 0, 0.5, 1.5, and 2.5% of PEG-DA, respectively. Mean ± SD: *n* = 3/group. Analysis revealed significant effects of cross-linking by the addition of PEG-DA on the swelling ratio (^*^ *P* < 0.05).

The addition of PEG-DA cross-linker significantly decreased the swelling ratio (*P* < 0.05, power = 0.71), as shown in Figure 1. Notably, the swelling ratio decreased from 42 ± 7.5 in PF-0 to 29.3 ± 0.8 in PF-2.5 hydrogel. These results indicate that an inverse correlation exists between the amount of additional PEG-DA and the swelling ratio, whereby hydrogels with higher PEG-DA concentration induce less swelling due to higher network cross-linking degree with a smaller mesh size.

### 3.2. DPSC Viability and Cell Shape Changes in PF Hydrogels

**Figure 2:**
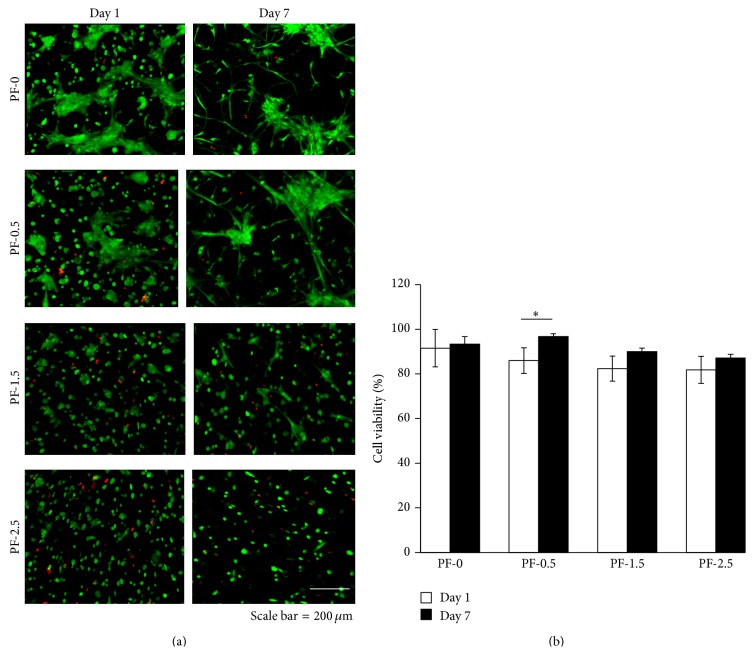
Cytocompatibility of PF hydrogels (PF-0, PF-0.5, PF-1.5, and PF-2.5) as a function of degree of cross-linking after 1 and 7 days of culture. (a) Representative live/dead images of DPSC-laden PF hydrogels at days 1 and 7 of culture, with live cells stained green and dead cells shown in red (*n* = 3/group/time point; scale bar = 200 *μ*m). (b) Percentage of cell viability of DPSCs at days 1 and 7 of culture. Mean ± SD: *n* = 3, 2-3 images/gel sample. ^*^ *P* < 0.05, Fisher's* post hoc* test.

DPSCs were encapsulated within PF hydrogels (PF-0, PF-0.5, PF-1.5, and PF-2.5) during photopolymerization to create 3D cell-seeded constructs. Cellular viability was assessed using live/dead staining after 1 day and 7 days in culture (Figure 2(a)). Viability, assessed by live/dead staining 1 day after encapsulation, indicated high viability (>90%) of DPSCs encapsulated in PF-0, with an observable trend of decreasing cellular viability with increasing percentage of PEG-DA cross-linker. Approximately 15% cell death was observed in PF-2.5 hydrogels (Figure 2(b)). By day 7 of culture, greater than 85% viability was observed in all hydrogels, although there seemed to have a slight decrease in cell number, possibly due to cell aggregation.

**Figure 3:**
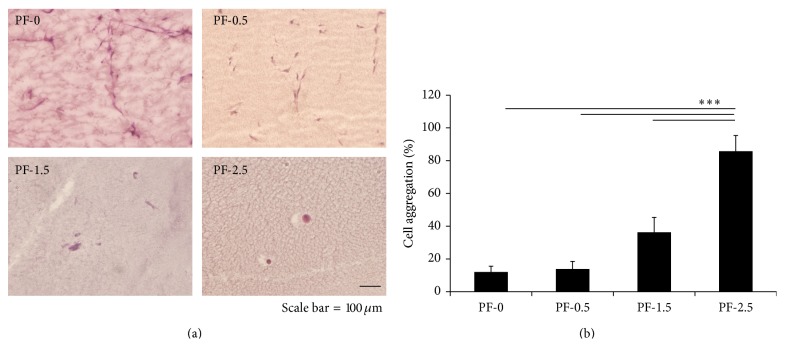
Effect of hydrogel degree of cross-linking on morphology of encapsulated DPSCs. (a) Representative images of DPSC-laden PF hydrogels following 21 days of culture (*n* = 3; scale bar = 100 *μ*m). (b) Percentage of cell aggregation after 21 days of culture. Mean ± SD: *n* = 3, 2-3 images/gel sample. ^***^ *P* < 0.001, Fisher's* post hoc* test.

In PF-0 and PF-0.5 hydrogels, cells were initially rounded but rapidly became spindled within 24 h (Figure 2(a)). By day 7 in culture, most of the DPSCs became highly spindled. A small number of cell clusters with spindle-like cytoplasmic extensions could also be observed. Notably, DPSCs remained spindled in PF-0 and PF-0.5 hydrogels for the duration of time in culture (Figure 3). Conversely, in PF-1.5 and PF-2.5 hydrogels, DPSCs exhibited fewer cell extensions and a decreased spindled morphology. Evidently, as revealed in the histological cross-sections of the gel constructs, cells were less able to form extensions in the dense polymer network containing the additional PEG-DA cross-linker (PF-1.5 and PF-2.5) and remained mostly rounded in aggregates for the duration of 21-day culture (Figure 3(a)). Quantitative analysis further indicated a relationship between the degree of cross-linking and the extent of cell aggregation (Figure 3(b)). By the end of 21-day culture, the highest cross-linked PF-2.5 hydrogels exhibited the highest percentage of cell aggregation (88.6 ± 9.8%). With increase in gel cross-linking, there was a significant increase in percentage of cell aggregates (Figure 3(b)). One-factor ANOVA revealed significant effect of gel cross-linking on the extent of cell aggregation (*P* < 0.0001, power = 1).

### 3.3. DPSC Differentiation and Mineralization in PF Hydrogels

**Figure 4:**
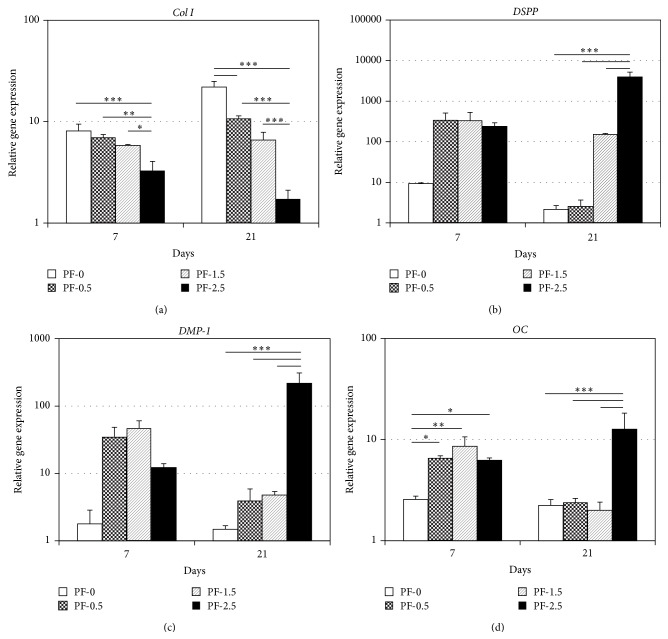
Odontogenic differentiation of encapsulated DPSCs in PF hydrogels (PF-0, PF-0.5, PF-1.5, and PF-2.5) as a function of degree of cross-linking and culture time. Relative gene expression levels of* Col I*,* DSPP*,* DMP-1*, and* OC* were determined with respect to the day 0 expression level and presented as bar graphs using logarithmic scales. Mean ± SD: *n* = 3/group/time point. ^*^ *P* < 0.05, ^**^ *P* < 0.01, and ^***^ *P* < 0.001, Fisher's* post hoc* test.

The effect of the mechanical properties of the tunable 3D PF hydrogels on the differentiation of DPSCs under odontogenic conditions over a 21-day time course was assessed (Figure 4). The expression levels of genes related to the odontogenic differentiation of DPSCs, including* Col I*,* DSPP*,* DMP-1,* and* OC*, were measured using quantitative real-time RT-PCR at days 7 and 21 of differentiation (Figure 4). The expression level of* Col I* gene was highest in PF-0 hydrogels at day 7 of differentiation and increased with culture time. By day 21 of differentiation, gene expression level of* Col I* in PF-0 hydrogel was approximately 13-fold higher than that in PF-2.5 hydrogel (Figure 4(a); *P* < 0.001). By contrast, the expression level of* Col I* gene was consistently the lowest in PF-2.5 hydrogels at different time points (Figure 4(a)). There were no significant differences in gene expression levels of* DSPP* and* DMP-1* among the PF hydrogels at day 7 of differentiation (Figures 4(b) and 4(c)). However, by day 21 of differentiation, the gene expression levels of* DSPP* and* DMP-1* increased in PF hydrogels with higher % PEG-DA and were the highest in PF-2.5 hydrogels (Figures 4(b) and 4(c)). Notably, gene expression levels of* DSPP* and* DMP-1* in PF-2.5 hydrogels were approximately 1800-fold (*P* < 0.001) and 150-fold (*P* < 0.001) higher than that in PF-0 hydrogels, respectively. The gene expression levels of* OC* were higher in PF hydrogels with added PEG-DA (0.5, 1.5, and 2.5%) at day 7 of differentiation (Figure 4(d)). However, by day 21 of differentiation, the gene expression levels of* OC* in PF-0.5 and PF-1.5 hydrogels decreased to basal levels comparable to that in PF-0 hydrogel, while gene expression level of* OC* in PF-2.5 increased with culture time. Notably, the gene expression of* OC* in PF-2.5 hydrogel was at least 5-fold higher than the other PF hydrogels (Figure 4(d); *P* < 0.001).

**Figure 5:**
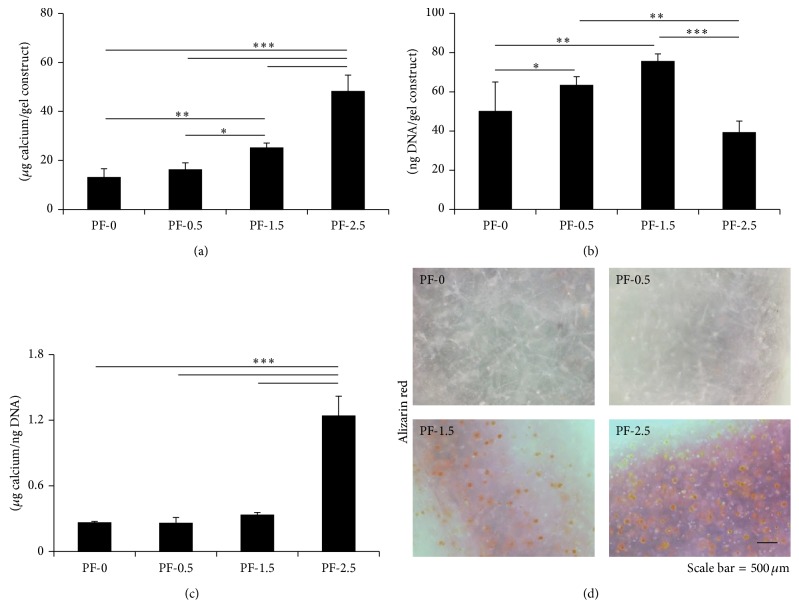
Calcium quantification of encapsulated DPSCs in PF hydrogels (PF-0, PF-0.5, PF-1.5, and PF-2.5) as a function of degree of cross-linking after 21 days of culture. (a) Total calcium content per gel construct. (b) DNA content per gel construct. (c) Calcium content per cell based on the amount of calcium normalized by the DNA content in the construct. Mean ± SD: *n* = 3. (d) Alizarin red staining of the DPSC-laden gel constructs. Red color indicates calcium deposition. Scale bar = 500 *μ*m. ^*^ *P* < 0.05, ^**^ *P* < 0.01, and ^***^ *P* < 0.001, Fisher's* post hoc* test.

The mineralization in hydrogel scaffolds was examined using Alizarin red staining and calcium quantitative assay (Figure 5). Calcium quantification revealed significantly higher levels of calcium deposition in PF hydrogels with higher % PEG-DA (Figure 5(a)). One-factor ANOVA revealed significant effect of gel cross-linking on the extent of calcium deposition (*P* < 0.0001, power = 1). By the end of 21-day differentiation, the DNA content (number of DPSCs) was the lowest in the highest cross-linked PF-2.5 hydrogel (Figure 5(b)). When normalized by the DNA content, highest level of calcium content on a per cell basis was observed in PF-2.5 hydrogel, with a value of at least 3.5-fold higher than the values for the other PF hydrogels (Figure 5(c); *P* < 0.001). This result was further confirmed by Alizarin red staining (Figure 5(d)) that observed mineralization only in PF-1.5 and PF-2.5 hydrogels, where cells remained mostly rounded in aggregates. In contrast, no mineralization was observed in PF-0 and PF-0.5, where most cells remained spindled.

## 4. Discussion

The use of injectable and* in situ* gel-forming systems is particularly attractive for clinical translation of dental pulp regeneration, as they can be easily formulated with growth factors and cells by simple mixing and can conform to the variable shape of the pulp chamber, following injection. Therefore, there has been a surge of interest in the development of injectable hydrogels in recent years for tissue engineering [20], particularly dental pulp tissue engineering [8–12].

In the design of the scaffold for dental pulp tissue engineering, several parameters including the matrix composition and architecture have been reported to influence adhesion, proliferation, and differentiation of dental stem cells and progenitors. While most studies [11, 12, 16] focus on modification of the scaffold to enhance odontogenic differentiation and biomineralization, the influence of matrix stiffness on the differentiation of DPSCs is still largely unclear. Separately, several studies have reported the influence of matrix stiffness on modulation of cell fate of a wide variety of cell types including stem cells [21–26]. In particular, in a landmark study, Engler et al. demonstrated that the elasticity of the matrix influences the differentiation of MSCs into neurons, myoblasts, and osteoblasts in ascending order of stiffness, with the stiffest matrices supporting MSC differentiation to osteoblasts [21]. Thus, it is the goal of this study to investigate the effects of varying hydrogel cross-linking degrees and corresponding matrix stiffness on the differentiation of DPSCs using a tunable hydrogel system.

Previous studies utilizing natural biopolymer gels (collagen, Matrigel, PuraMatrix, and hyaluronic acid) do not provide a means to tune the mechanical properties independently from matrix composition that confers biofunctional properties of adhesion and proteolytic sensitivity [8, 9, 12]. The semisynthetic PEG-fibrinogen (PF) hydrogel was particularly useful for this study because the mechanical properties are tunable by the addition of cross-linker (PEG-DA) that controls the hydrogel cross-linking degree, while maintaining a constant fibrinogen backbone. This unique feature allows us to investigate the effects of mechanical properties of hydrogel on DPSC differentiation systematically and independently.

In general, PF hydrogels were cytocompatible with DPSCs, although there was a slight decrease in cell viability with increasing cross-linking degree and matrix stiffness. Distinct differences in cell morphology of DPSCs could be observed in PF hydrogels with varying cross-linking degrees. Evidently, cells formed clusters with spindle-like cytoplasmic extensions within the lower cross-linked PF hydrogels (PF-0 and PF-0.5), which is frequently observed as a common feature of these cells in soft matrices such as the PuraMatrix [8, 9]. The ability to spread and elongate may also relate to the ability of the cells to secrete fibrinolytic enzymes to break apart the hydrogel network more readily in the lower cross-linked hydrogels than in the higher cross-linked counterparts [27]. Conversely, cells tended to be rounded and forming aggregates confined within the dense polymer network of the higher cross-linked hydrogels (PF-1.5 and PF-2.5). It is well known that cell morphology and function are tightly coupled [28]. Notably, lower cross-linked matrices that induced spindle-like elongation of DPSCs resulted in more pronounced gene expression of* Col I*. Although* Col I* is one of the extracellular matrix (ECM) components of the demineralized dentin, it is also a major collagenous ECM protein present in many other tissues. With the concomitant upregulation in* Col I* but downregulation of other odontogenic markers including* DSPP*,* DMP-1*, and* OC*, there is likelihood that DPSCs were guided by the softer matrices to differentiate to lineages other than the odontoblasts and osteoblasts.

On the other hand, the higher cross-linked stiffer hydrogels (PF-1.5 and PF-2.5) tested in this study, with a *G*′ ranging from 1500 to 3600 Pa, generally favored odontoblastic differentiation of DPSCs. The PF-2.5 hydrogel constructs at a stiffness of *G*′ ~3600 Pa induced the highest gene expression of* DSPP*,* DMP-1*, and* OC* by the end of 21-day differentiation. It is likely that the heightened odontogenic/osteogenic differentiation is a result of the DPSCs forming cell aggregates and mechanosensing the stiffer matrix that has been shown in previous studies to promote stem cell osteogenesis [29, 30]. Consistent with the gene expression results, mineralization was only observed in higher cross-linked PF-1.5 and PF-2.5 hydrogels, with the latter showing the most robust mineralization. Of interest, there was a strong correlation between cellular aggregation denoted by the percentage of rounded cells and the extent of calcium deposition (linear regression analysis; *R* ^2^ > 0.92).

With the lowest relative cell number by the end of 21-day differentiation, it is likely that cellular aggregation observed in the highest cross-linked PF-2.5 hydrogel promoted odontogenic differentiation but lesser extent of proliferation. Nevertheless, a more thorough and quantitative assessment would be necessary for a more complete characterization of the cellular proliferation during the course of differentiation.

Collectively, the injectable PF hydrogels are cytocompatible with DPSCs, and the hydrogel mechanical properties (i.e., cross-linking degree and matrix stiffness) and biofunctional properties conferred by fibrinogen could be tuned to provide a supportive biomimetic cellular microenvironment for odontogenic differentiation. These results suggest a possible use of these hydrogels as scaffolds for dentin-pulp tissue engineering and regeneration. To the best of our knowledge, this work represents the first demonstration of the influence of matrix stiffness on DPSC odontogenic differentiation in 3D tunable hydrogels. Further studies through tooth slice organ culture [31] and* in vivo* transplantation studies [9, 32] would be needed to assess if these hydrogels are supportive of the formation of new tubular dentin and pulp tissue complex for dental pulp regeneration.

## 5. Conclusions

Injectable,* in situ* forming hydrogels are particularly attractive for dental pulp tissue engineering, as they can be easily formulated with growth factors and cells by simple mixing, and can conform to the pulp chamber following injection. In this study, we investigated the use of injectable PF hydrogels as scaffold carriers for DPSCs for dental pulp tissue engineering and provided strong evidence that the tunable 3D microenvironment of the PF-hydrogels modulates odontogenic differentiation and mineralization of human DPSCs.

## Figures and Tables

**Table 1:** List of primers.

Target	Forward	Reverse
*COL I *	AAAAGGAAGCTTGGTCCACT	GTGTGGAGAAAGGAGCAGAA
*DSPP *	TTAAATGCCAGTGGAACCAT	ATTCCCTTCTCCCTTGTGAC
*DMP-1 *	TGGGGATTATCCTGTGCTCT	TACTTCTGGGGTCACTGTCG
*OC *	CATGAGAGCCCTCACA	AGAGCGACACCCTAGAC
*GAPDH *	GAGTCAACGGATTTGGTCGT	GACAAGCTTCCCGTTCTGAG

**Table 2:** Characterization of PEG-fibrinogen (PF) hydrogels^a^.

Gel type	% PEG-DA	*G*′ (Pa)	Gel point (s)^b^	Time required to reach *G*′ plateau (s)
PF-0	0	140.1 ± 1.8	11.4 ± 0.2	38.2 ± 0.2
PF-0.5	0.5	453.8 ± 12.6	11.5 ± 0.1	51.7 ± 0.1
PF-1.5	1.5	1574 ± 21.2	17.4 ± 2.6	82.6 ± 0.1
PF-2.5	2.5	3601 ± 46.5	23.4 ± 2.5	127.4 ± 4.5

^a^Measurements were taken at room temperature in the time-sweep oscillatory mode with a sinusoidal 2% strain rate and a 6 rad s^−1^ angular frequency (Mean ± SD; *n* = 3).

^b^Gel point is defined as the time at which the crossover of storage modulus (*G*′) and loss modulus (*G*′′) occurred. Herein, it is used as an indicator of the rate of gelation.
